# 

**Published:** 1879-11

**Authors:** 


CONTENTS:

ARTICLE I.—The Progress of Dentistry. By Richard Grady, D. D. S. .......... 289
ARTICLE II.—Transactions of the Odontological Society of Great Britain.......... 299
ARTICLE III.—Proceedings of the Maryland and District of Columbia Dental Society.. 305
ARTICLE IV.—Lecture on the Morphology of the Blood in Syphilis. By Ephraim Cutter, M. D.......... 317

EDITORIAL, ETC.

Terrible Case of Arsenical Poisoning.......... 328
Supply—Demand.......... 331

BIBLIOGRAPHICAL.

The Origin and Formation of the Dental Follicle.......... 332
The American Agriculturist.......... 333
Winter and its Dangers.......... 334
St. Maur; An Earl's Wooing.......... 334

MONTHLY SUMMARY

Nitrous Oxide in Asthma.......... 335
Copperas and other Disinfectants.......... 336
Dental Caries.......... 336

